# New Tools for Molecular Therapy of Hepatocellular Carcinoma

**DOI:** 10.3390/diseases3040325

**Published:** 2015-10-30

**Authors:** Alessandra Marchetti, Francesca Bisceglia, Angela M. Cozzolino, Marco Tripodi

**Affiliations:** 1 Department of Cellular Biotechnologies and Hematology, Pasteur Institute-Cenci Bolognetti Foundation, Sapienza University of Rome, Viale Regina Elena, 324, 00161 Rome, Italy; E-Mails: bisceglia@bce.uniroma1.it (F.B.); cozzolino@bce.uniroma1.it (A.M.C.); 2 National Institute for Infectious Diseases Lazzaro Spallanzani, IRCCS, Via Portuense, 292, 00149 Rome, Italy

**Keywords:** HCC, HNF4α, LETFs, miRNAs, TGFβ, EMT, gene therapy

## Abstract

Hepatocellular carcinoma (HCC) is the most common type of liver cancer, arising from neoplastic transformation of hepatocytes or liver precursor/stem cells. HCC is often associated with pre-existing chronic liver pathologies of different origin (mainly subsequent to HBV and HCV infections), such as fibrosis or cirrhosis. Current therapies are essentially still ineffective, due both to the tumor heterogeneity and the frequent late diagnosis, making necessary the creation of new therapeutic strategies to inhibit tumor onset and progression and improve the survival of patients. A promising strategy for treatment of HCC is the targeted molecular therapy based on the restoration of tumor suppressor proteins lost during neoplastic transformation. In particular, the delivery of master genes of epithelial/hepatocyte differentiation, able to trigger an extensive reprogramming of gene expression, could allow the induction of an efficient antitumor response through the simultaneous adjustment of multiple genetic/epigenetic alterations contributing to tumor development. Here, we report recent literature data supporting the use of members of the liver enriched transcription factor (LETF) family, in particular HNF4α, as tools for gene therapy of HCC.

## 1. Introduction

Hepatocellular carcinoma (HCC) is one of the most common cancers worldwide and the most frequent among the primary tumors of the liver. HCCs are phenotypically and genetically heterogeneous tumors, since they often develop on the pathological background of pre-existing chronic liver diseases, including fibrosis or cirrhosis in consequence of HBV and HCV infections, alcoholic injury, or autoimmune hepatitis, that impair organ function and reduce the efficacy of common cancer therapies [1]. Moreover, most HCC patients are diagnosed at advanced stages of disease when the high tumor recurrence rate and the tendency to metastasize make current treatments ineffective and the prognosis poor [2].

In recent years, intense pre-clinical and clinical research have been devoted to the development of tailored therapeutic molecules, capable of restoring the physiological cell functions lost in transformed hepatocytes, through the gene therapy of HCCs. Gene therapy is a promising approach, since it is possible to deliver vectors directly into hepatic tumors, reducing potential side effects derived from transduction in non-target cells. Molecules utilized in current protocols include genes for proteins or microRNAs (miRNAs) displaying antitumor properties (anti-proliferative, pro-apoptotic, anti-angiogenic, or immunomodulatory).

Unfortunately, highly effective results have not been obtained so far, due to the low efficiency of the gene transfer [3] and to the genetic heterogeneity of HCCs [4]. For this reason, the most promising candidates would be oncosuppressor genes able to induce an efficient antitumor response without a specific correction of multiple mutations contributing to tumor development (e.g., p53) or differentiation-specific master genes, able to act as reprogramming transcriptional factors, coordinating extensive gene expression. In the context of the latter strategy, we will discuss recent progress in the knowledge of HCC biology and genetics supporting the use of Liver Enriched Transcription Factors (LETFs), and in particular of hepatocyte nuclear factor 4α (HNF4α), as promising candidates for targeted gene therapy of HCCs.

## 2. Molecular Alterations in HCC

### 2.1. Cell-Autonomous Changes

In spite of the heterogeneity of HCC, tumor onset and progression have been associated with recurring cell-autonomous molecular changes [5] such as the loss of expression of differentiation genes (e.g., Hepatocyte Nuclear Factor 1 and 4, HNF1α, and HNF4α) [6,7], chromosomal instability leading either to the loss of heterozygosity in tumor suppressor genes (e.g., p. 53) [8] or to the amplification of loci for oncogenes (e.g., ERK5) [9], and aberrant activation of signaling pathways (e.g., Wnt/β-catenin pathway) [10].

In addition, recent findings highlight how epigenetic alterations are commonly observed in human HCCs [11,12] and can be exploited as clinical predictors for diagnosis and prognosis [13]. These alterations include aberrant methylation of tumor suppressor genes [13], post-translational histone modifications [14,15], and altered expression profile of miRNAs [16,17].

The progression of HCC toward more aggressive stages, responsible for the worst prognosis in patients, is frequently associated to the activation, in transformed hepatocytes, of a transdifferentiation process: the epithelial-to-mesenchymal transition (EMT). EMT contributes to tumor progression through the loss of epithelial/hepatocyte cell differentiation, the acquisition of motility/invasivity properties and cancer stem cell traits, the resistance to apoptosis, and metastasis (reviewed in [18]). Overexpression of EMT markers (*i.e*., Snail and Twist) has been reported in invasive areas of primary tumors [19] and in metastasis of aggressive hepatocarcinomas [20], and were associated with poor prognosis. Their analysis in circulating tumor cells has been recently proposed as prognostic tools for HCC patients [21]. The role of EMT master genes, in particular Snail, in tumor progression was found to be mediated by (i) the direct transcriptional repression of an extensive amount of target genes involved both in epithelial (e.g., E-cadherin) [22] and hepatic (e.g., HNF4α) [23] differentiation, (ii) the increase of mesenchymal gene expression [23], and iii) the miRNA-mediated up-regulation of stemness genes [24]. The acquisition of EMT-related stem cell characteristics has been demonstrated to positively correlate with HCC progression [25]. The presence of stemness traits in HCC tumor cells, indeed, has been associated with chemoresistance and tumor recurrence after surgery [26,27] and can contribute to the intratumoral heterogeneity of HCC tumor cells [28].

### 2.2. Non-Cell-Autonomous Cues

An important role in HCC is also played by non-cell-autonomous cues, such as the presence of factors in the tumor niche promoting tumor growth or influencing proliferation/activation of tumor-associated fibroblasts [29,30].

In particular, the role of TGFβ cytokine in the progression of HCC was largely described. In HCC patients elevated plasma levels of TGFβ have been reported, correlating with poor prognosis [31,32]. Furthermore, in late stage HCC, the TGFβ signaling pathway is constitutively activated [33] and is involved in promoting tumor invasion through stimulation of vascularization [34], acquisition of stem-like features [35], and induction of EMT [36]. The molecular signature of late stage HCC-derived cell lines, indeed, showed high levels of EMT markers (including matrix metalloproteases, vimentin and, particularly, Snail) and down-regulation of genes for liver-specific functions, indicating a reduced hepatocyte/epithelial differentiation state [37]. TGFβ signaling, moreover, can be amplified in HBV-infected HCC cells by HBx protein [38].

TGFβ signaling is also closely linked to liver diseases favoring development of HCC. TGFβ can activate hepatic stellate cells (HSCs) [39] and induce an immune response, causing fibrosis and leading to HCC onset [40]. Its serum levels are also associated to virus-induced fibrosis [41,42]. Furthermore, TGFβ can induce production of CTGF (Connective Tissue Growth Factor) by cancer-associated fibroblasts promoting tumor progression [43].

Recent reports highlighted a role of biophysical changes in extra-cellular matrix stiffness as microenvironmental cues influencing tumor growth and progression. Fibro-cirrhotic livers, for example, are characterized by a significant increase of ECM stiffness [44]. YAP/TAZ were recently identified as molecular relay of mechanical stimuli exerted by ECM stiffness, inhibited by the Hippo signaling pathway and involved in organ size control [45]. Dysregulation of the Hippo/YAP cascade has been recently reported for several human tumors, including HCC, and correlates with increased cell proliferation and survival, acquisition of stemness properties, and metastasis (reviewed in [46]). In particular, YAP overexpression was found in human HCC samples [47,48] and correlates with poor prognosis of HCC patients [49]. Furthermore, its inhibition in cells from advanced HCC restores hepatocyte differentiation inducing the up-regulation of master factors (*i.e.*, HNF4α/FOXA1/FOXA3) and leading to tumor regression [50]. Interestingly, YAP protein is directly involved in switching occupancy of HNF4α on embryonic hepatoblast genes to adult hepatocyte genes [51], suggesting a direct role of YAP in influencing the function of key transcriptional factors and master genes of hepatocyte differentiation.

## 3. Gene Therapy of HCC: Is It a Good Deal?

As highlighted above, current therapies for HCC are still ineffective. Surgical liver resection efficiency is limited to small localized tumors with low risk of recurrence in non-cirrhotic patients. Conventional chemotherapy is largely unsuccessful due to tumor cell resistance and side effects of “non-selective” cytotoxic drugs. Furthermore, the immunosuppression associated to HCC (mainly subsequent to chronic HBV and HCV infections) negatively impacts on tumor recurrence. For this reason, immuno-based therapies have been proposed to ameliorate the clinical outcome of HCC patients (reviewed in [52]).

Targeted approaches have also been applied, in particular for advance-stage and unresectable HCC. These treatments include oral administration of the multikinase inhibitor sorafenib, or single target agents, such as gefitinib and erlotinib, currently involved in ongoing clinical trials in the US and EU [53]. The therapy with sorafenib, in particular, showed prolonged median overall survival and delayed the median time to progression in patients with HCC, showing limited and manageable adverse effects [54]. However, chronic liver diseases that usually underlie HCC may enhance the hepatotoxicity of these agents; accordingly, the prognosis of late-stage HCC patients is still poor [53].

In this context, the targeted gene therapy for the management of HCC seems to be the most promising approach. In particular, the adenoviral mediated gene therapy is well documented and included in several human clinical trials where the tolerance is high and side effects acceptable in most of the cases (reviewed in [55]). However, the efficiency of transduction and the tumor specificity still remain limiting factors for this approach. In HCC, these problems could be overcome by intratumoral administration of vectors and/or by the use of tumor-specific promoters that may restrict the delivery to hepatocytes (e.g., AFP) [56], especially improving efficacy and minimizing the toxicity of this therapeutical strategy.

Among different approaches of gene therapy (restoration of oncosuppressors, delivery of suicide genes, or inhibition of oncogenes) the delivery of “differentiating” factors could achieve the best results in terms of low toxicity and maintenance of tissue homeostasis, especially compared to killing drugs or agents inducing apoptosis. The most severe consequence may be related to the damage of the stem cell compartment with the decreased number of cells (stem cells or progenitors) responsible for tissue renewal. However, in the liver, the real involvement of resident liver stem/precursor cells in hepatic regeneration after chronic injury is strongly debated since it has been recently formally proved that adult hepatocytes originate from self-duplication of other hepatocytes rather than from stem cell differentiation [57,58]. Altogether, this knowledge suggests that the ectopic expression of differentiation master genes in the liver could be tolerated and the side effects reduced.

## 4. LETFs as Molecular Tools for Gene Therapy of HCC

Maintenance of hepatocyte differentiation and control of liver-specific gene expression are attributed in large part to hepatocyte nuclear factors (HNFs) belonging to the LETF family, including HNF1α, HNF4α, HNF6, and FOXA2 [59]. Being reciprocal transcriptional activators, they operate cooperatively in a connected network in the liver, regulating several developmental and metabolic functions in hepatocytes [60,61].

### 4.1. HNF4α

The nuclear receptor HNF4α is a key regulator of hepatocyte differentiation during embryonic development [62,63], influencing the expression of other hepatic transcription factors, and stabilizing co-regulatory networks for the maintenance of a differentiated phenotype [61]. In the adult liver, HNF4α is highly expressed in hepatocytes. HNF4α maintains hepatocyte identity both by inducing epithelial/hepatic differentiation through a direct regulation of epithelial and metabolic target genes [62,64], and by actively inhibiting mesenchymal differentiation program through a direct repression of mesenchymal and EMT master genes [65]. Accordingly, experimental HNF4α deletion in adult mouse livers has been shown to lead to dedifferentiation and proliferation of hepatocytes, hepatomegaly, and expansion of precursor cells (*i.e.*, oval cells) [66,67].

HNF4α is a strong inducer of mesenchymal-to-epithelial transition (MET). Its ectopic expression in fibroblast [62] and F9 cells [68] is sufficient to trigger epithelial gene expression and acquisition of epithelial polarity. Furthermore, HNF4α, together with FOXA1, FOXA2, or FOXA3, was found capable of inducing the direct reprogramming of mouse fibroblasts into hepatocyte-like cells [69].

Importantly, in addition to the transcriptional regulation of mRNAs, HNF4α regulates the expression of miRNAs which, in turn, can act as pleiotropic elements influencing differentiation, EMT, stemness, and hepatocarcinogenesis.

In particular, HNF4α (as well as other LETFs) was found to directly regulate expression of the liver-specific microRNA-122 (miR-122) [70], the most abundant miRNA in hepatocytes, and the first miRNA suggested as a tumor suppressor in the liver. Its expression, indeed, is frequently reduced in HCCs [71] and is associated with low differentiation, migration/invasivity of HCC cells [72,73], and poor prognosis in patients [72]. Mir-122 restoration in HCC cells leads to a reduction of mesenchymal markers [74], cell-cycle arrest or apoptosis [75], and sensitizes cells to antitumor agents [76,77]. Notably, miRNA-122 delivery in HCC murine models impaired tumor occurrence, growth, and progression [73,78].

Recently, the transcriptional regulation of other miRNAs, *i.e.*, members of the miR-200 family and miR-34a, by HNF4α has been described and showed to contribute to the active repression of stem cell genes [24]. Both miR-200 family members and miR-34a were suggested to function as tumor suppressors in HCCs. They appeared markedly down-regulated in HCC [79,80] and their restoration in various cancer stem cells is associated with the loss of stem cell traits, inhibition of EMT, cell differentiation, and decreased motility/invasivity [24,81,82]. However, the role of miR-34a in cancer is currently debated [83] and, in HCC, has been related to the cellular context [84].

It has been recently shown that HNF4α controls the epigenetic state of differentiated hepatocytes through the miR-29-mediated DNMT3A,B down-regulation [85]. Interestingly, low levels of miR-29 and DNMT3A,B up-regulation correlate with TGFβ-induced EMT, liver fibrosis, and aggressiveness of HCC [86,87,88]. Being the epigenetic changes, including DNA methylation, sustained by the presence of high levels of DNMTs, involved in both EMT [89] and hepatocarcinogenesis [90], miR-29 could represent a good target for a therapeutic approach aimed at the epigenetic reprogramming of HCC cells.

Several lines of evidence indicate HNF4α as a potential tumor suppressor of HCC. In mature hepatocytes, loss/inactivation of its function resulted in an increased risk for development of HCC. Transient inhibition of HNF4α is sufficient to initiate hepatocellular transformation in non-transformed hepatocytes and to increase invasiveness in transformed HCC cell lines through a microRNA-mediated inflammatory loop circuit [91]. This network can also contribute to the maintenance of HNF4α inactivation during hepatocellular transformation [91]. Several studies have shown a decreased expression of HNF4α in both murine models and human samples of HCC, thus indicating a critical role of this protein in the HCC onset/progression [7,92,93]. As a consequence, the restoration of HNF4α expression/function in HCCs has represented, in the last few years, an important goal for molecular approaches to HCC treatment. The whole described tumor-suppressing functions of HNF4α indicate that this protein represents a good candidate for the extensive reprogramming of tumor cells and, therefore, a promising tool for gene therapy of HCC.

Several data substantiate this expectation. Forced HNF4α expression in dedifferentiated and aggressive HCC is sufficient to reduce tumor cell motility/invasivity by inducing differentiation and EMT inhibition [65,92]. Moreover, HNF4α overexpression attenuates hepatic fibrosis and, in fibrotic livers, can prevent HCC occurrence by blocking the activation of myofibroblasts [93,94]. Furthermore, overexpression of HNF4α in rodent HCC models blocks carcinogenesis and metastasis [93,95].

Overall, the restoration of the HNF4α functions in invasive HCCs has been proven to be an efficient approach for the gene therapy of HCC, at least in experimental models. However, recent data have shown how microenvironment cues could reduce the efficacy of this approach. In particular, the presence of TGFβ in the tumor niche impaired HNF4α activity by inducing the displacement of the ectopic protein from its target gene promoters through the inactivation of GSK-3β activity [96]. This result suggests the need to obtain improved HNF4α proteins as tools for gene therapy, through the design of TGFβ-insensitive mutants.

At the same time, the potential tumor suppressor activity of other members of the LETF family should be explored. Recently, the role in tumor suppression of HNF1α and HNF6, has been described. Similarly to HNF4α, indeed, these proteins are down-regulated in HCC and their overexpression in tumor cell lines was found to suppress EMT and invasion [92].

### 4.2. HNF1α

HNF1α is a homeodomain protein that plays a critical role in hepatocyte differentiation. It contributes to the expression of products central in normal hepatic functions [97] and is required for the maintenance of the differentiated state [98]. HNF1α, moreover, together with HNF4α and HNF6, leads to the generation of functional human-induced hepatocytes (hiHeps) from fibroblasts [99] and its overexpression is necessary for the direct reprogramming of human fibroblasts to hepatocyte-like cells [100].

Extensive evidence suggested that HNF1α acts as a tumor suppressor gene and that its down-regulation contributes to the development of HCC. HNF1α gene was found mutated in 84% of cases of adenomas, including familial forms [101,102], and HNF1α protein levels were found significantly reduced in moderately- and poorly-differentiated tissues from HCCs [6]. Furthermore, HNF1α knock-out mice exhibit tumor-associated characteristics, such as increased proliferation of hepatocytes, leading to a dramatic liver enlargement and liver function defects [103].

Taken together, these findings suggested that restoration of HNF1α functions in HCC could restrain tumor proliferation and progression. Zeng *et al.* recently demonstrated that the forced re-expression of HNF1α in human hepatoma cell lines induces a re-establishment of hepatic differentiation through the significant induction of liver specific genes and the repression of cell proliferation. Most importantly, intratumoral HNF1α transduction significantly inhibits tumor growth in mice and eradicates HCC nodules after systemic delivery [104].

HNF1α is not only a promising therapeutic tool for a differentiation therapy in HCC treatment but it could be also a potent anti-EMT tool, being a strong transcriptional repressor of EMT master genes as HNF4α [65]. Accordingly, suppression of HNF1α in HCC cell lines triggers expression of mesenchymal and EMT master genes, overexpression of TGFβ, and migration [105].

### 4.3. HNF6/ONECUT1

HNF6 represents another potential molecular tool for tumor suppression in HCC. It is essential for expression of hepatic genes, also controlling the direct expression of HNF4α [106] and genes involved in glucose metabolism [107,108]. Moreover, HNF6 synergistically cooperates with HNF4α and HNF1α for the regulation of hepatocyte differentiation during development and in the adult. HNF6 is also a strong transcriptional activator of miR-122, establishing a positive feedback loop responsible for *in vivo* hepatocytes differentiation [109] that may contribute to prevent neoplastic transformation.

HNF6, as well as other LETFs, is involved both in the maintenance of the epithelial differentiation and in the active repression of EMT program through the up-regulation of p53 tumor suppressor [110]. Furthermore, it is implicated in the inhibition of HCC progression [92].

HNF6 overexpression reduced the proliferation of liver cancer cell lines [111], inhibited colony formation and cell proliferation/migration in carcinoma cells, and decreased the formation of tumors in nude mice [110]. Conversely, knockdown of HNF6 induced EMT and increased cell migration [110]. Furthermore, HNF6 has been recently shown to interfere, *in vitro* and *in vivo*, with HBV infection through the inhibition of viral gene expression and DNA replication [112].

Notably, a potential inhibitory effect of HNF6 on TGFβ signaling has recently been reported. Components of TGFβ signaling pathway were activated in HNF6 knockout mice, at least in part, through the up-regulation of TGFβRII expression [113]. Interestingly, through the inhibition of TGFβ/activin signaling, HNF6 allows differentiation of precursor cells in hepatocytes [114]. These data, in light of what was previously observed for HNF4α, could indicate HNF6 as a more efficient tumor suppressor in the presence of TGFβ in the tumor microenvironment.

## 5. Conclusions

The unsuccessful therapeutic approaches for the treatment of HCCs lead to focus the attention on molecular strategies consisting of the intra-tumoral delivery of specific proteins with tumor suppressor properties.

**Figure 1:**
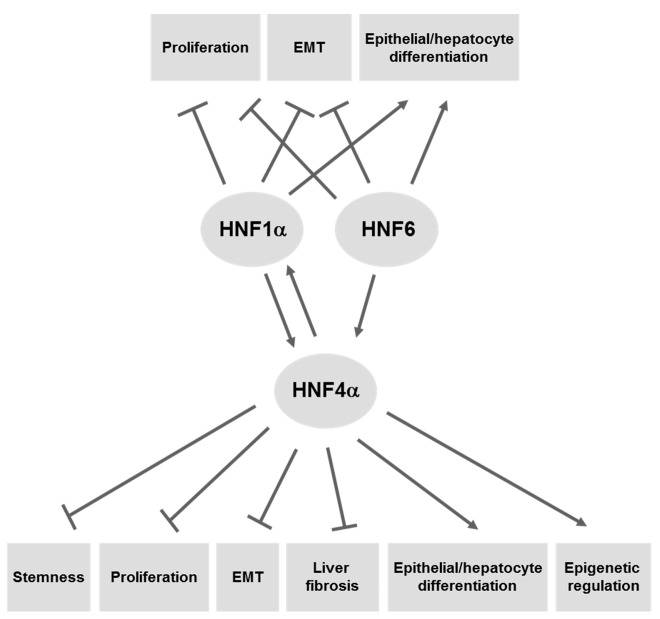
Tumor suppressor properties of HNF4α, HNF1α, and HNF6 in HCCs. HNFs can regulate different cell functions associated with the HCC onset and progression, through the direct transcriptional activation/repression of target genes (described in the text). The reciprocal regulation among HNFs is shown.

Recent literature data discussed above demonstrates the high potential of anti-cancer therapy based on the restoration of functions of epithelial/hepatocyte differentiation master regulators belonging to the LETF family, mainly HNF4α. These proteins are able to induce cellular reprogramming, coordinating extensive gene expression either through direct transcriptional regulation or by driving epigenetic changes on regulatory regions of target genes (Figure 1). LETFs, indeed, can not only induce the terminal differentiation of tumor cells (and potentially of cancer stem cells) but they can also interfere with the EMT program responsible for tumor progression. These characteristics make LETFs promising tools for molecular therapy of HCC. The challenge is now the optimization of these tools through the creation of engineered molecules to take in account the microenvironmental cues that could influence the effectiveness of this therapeutic approach. Further studies will be necessary to achieve this result.
